# Selection Process for Romanian Children in Handball Based on Coordinative Abilities: A Questionnaire Survey

**DOI:** 10.3390/ijerph19063672

**Published:** 2022-03-19

**Authors:** Ileana Petrariu, Florin Valentin Leuciuc

**Affiliations:** 1 Department of Physical Education and Sport, Stefan cel Mare University of Suceava, 13th University Street, 720229 Suceava, Romania; florin.leuciuc@usm.ro; 2 The Interdisciplinary Research Center for Human Motricity and Health Sciences, 13th University Street, 720229 Suceava, Romania

**Keywords:** coordinative abilities, training, handball, physical activity, opinion

## Abstract

According to the reviewed literature, the selection system in handball is quite complex and requires new approaches from trainers based on the current and modern requirements of this game. This paper investigated the importance of coordinative ability development in handball in the selection process for young Romanian children (10–12 years old). The results were obtained from a sociological questionnaire survey, for which 109 handball coaches, 34 female and 75 male, were questioned. The mean age was 40.71 years (SD = ±5.32) with a range of 31–51 years for female coaches and 40.3 years (SD = ±7.53) with a range of 30–62 years for male coaches. Their experience in handball coaching varied from 10 to 30 years, with 19.88 (SD = ±5.05) years of experience on average for female coaches and 19.09 (SD = ±5.26) years of experience on average for male coaches. The answers delivered depended on the experience and knowledge the coaches had and difficulties they had encountered over the years. Data are presented using a variety of appropriate descriptive statistics, including frequencies, percentages, and mean and standard variation. Obtained data were modeled using a one-way analysis of variation (ANOVA). The questionnaire format was three-to-five-point Likert scale. As a general overview, in the opinion of the surveyed specialists, the Romania selection process for handball does not meet their expectations, and the development level of coordinative abilities in 10–12 year-old children is medium to weak. A total of 61.76% female and 58.66% male coaches stressed the importance of developing coordination abilities to optimize the selection process of children for handball games, opening up a new approach in modern training methods for performance achievement. The obtained results from this paper questionnaire can be used by coaches as a different approach to the handball training process, considering that handball is a game mainly based on coordination.

## 1. Introduction

The need for thorough and forward-looking training of athletes is a major concern of researchers in terms of modern sports training nowadays. Training tomorrow’s athletes’ team is considered an important priority, and it is the main concern of the researchers. They try to make various contributions and improvements in sports training components based on the athletes’ age [1,2,3].

Currently, the selection process in Romania for handball games is carried out according to certain principles and rules that are no longer up to date, which determines a regression from a performance point of view.

The specialized literature is diversified in terms of selection issues but lacks the solutions that coaches are looking for. The harmonious combination of motor, morphological, psychological, and functional criteria, considering the optimal age of selection, health, and body integrity, is argued to improve handball performance.

Awareness of the importance of carrying out the selection process on a well-determined scientific basis, the constant drive to find specific innovative solutions, and the application of the criteria listed in optimal conditions provides the appropriate framework for initiation into sports activity with the aim to achieve good performance.

The main purpose of this study was to investigate handball coaches’ opinions and gauge to what extent they believe children’s coordinative abilities’ development may influence the quality of the selection process in Romanian handball. The study started due to the presumption that the development of children’s coordinative abilities is very important, especially since around the age of 7–12 years, they develop up to 75% of the maximum development potential reached in adolescence [4]. At the age of 10–12 years old, the development of coordination is intense, allowing the rapid acquisition of the technical–tactical elements of handball, improving the reaction ability in different situations of collaboration or adversity, developing the anticipation spirit, and space-time orientation. The emphasis on coordinative abilities’ development at this age is based on the plasticity of the nervous system in motor habits that are not permanently fixed, which can be corrected and perfected, making it easier for children to quickly learn this game.

The novelty and originality of this paper involves the use of a questionnaire survey instead of classical methods applied for collecting accurate results regarding the selection of children for handball. We used this approach because it is highly used in many research domains due to its easy process of collecting and processing valuable data. In sports, for example, Russell et al. [5] highlights that athletes experience development in relation to empirically identified “key features” of effective talent development environments, which can be designed to facilitate the development of sporting potential to a world-class standard, which can be analyzed through questionaries. Questionaries can also investigate psychological, social, physical, and environmental variables related to elite sports athletes [6]. The questionaries from [7,8] can assess sports nutrition knowledge (SNK), which is essential for athletes to optimize sport performance and to maintain a balanced and a healthy diet. Another questionnaire from [9] was used to assess psychological stress in elite athletes, a major factor that contributes to achieving sportive performance.

The obtained results from this paper’s questionnaire can be used by coaches as a different approach to the handball training process, considering that handball is a game mainly based on coordination.

## 2. Literature Overview

In team sports, situations, routines, strategies, and tactics occur, the knowledge of which is crucial to describe and analyze collective behavior [10], which is why coaches and researchers use various methods to examine and study players, teams, and their game to enhance the sports preparation process [11].

Handball involves a variety of unpredictable actions where the two teams act as a dynamical system. Regarding this method, the need to understand how players’ performance can contribute to obtaining greater scientific knowledge of technical actions emerges [12].

In many scientific papers, the optimal age to start training for different sports is discussed considering injury prevention for future young athletes [13,14]. For handball, Nabatnikova [15] and Manno [16] consider the optimal period for initial training to be between 10 and 13 years of age, followed by 12 to 17 years of age, for early specialization, which extends to 16 to 18 years of age for performance improvement. Baștiurea [17] supplements these above findings, including nontraditional training methods. They offer the possibility to individualize the training program of athletes, provide important information about the subject’s qualitative and quantitative parameters of motor and psychomotor actions, support the coach in activity planning during training, and are designed to teach the external characteristics of movement, without provoking the internal structure used for motor skills.

With the increase in sports performance, the content of sports training has become increasingly complex, resulting in changing methodology. This fact has led to significant changes in the sports training content systematization, the development of which follows some strict rules and principles. According to Shuba et al. [18], one of these rules aims to establish the purpose and the result of the physical exercise performed, a key element in sports training. Sports evolution is determined by the performance level. Thus, in sports, the body is trained to control its movements under unusual conditions, such as imbalances, jumps, or coordination, conditions that require a complex skill management system. Additionally, the same authors claim that the perceptual–motor learning process involves a change in the athlete’s behavior, depending on the unique conditions that occur in the training process or in sports competitions. Thus, the individual reacts with an answer that can be perfected in terms of coordination, accuracy, rhythm, etc. This reaction, which becomes a skill, is adjusted (through several interventions) until a perfect concordance is obtained between the anticipated image and the effective action. Thus, specific motor skills reach the final level of fluency, precision, coordination, and tempo through stereotypical repetitions.

At the FIMS World Congress of Sports Medicine [19], it was stated that to obtain sports performance, the process of selecting individuals has an importance of 70%; thus, 30% of the total is attributed to sports training. One can easily observe the major importance that researchers grant to the selection process, this being considered the key to performance in contemporary sport areas. As a general definition supported by other researchers [20,21,22], the selection process is a systematic and organized activity, a process of sorting and identification, carried out by coaches based on rigorously established criteria, to detect young children with special skills for practicing and specializing in a particular sport area to obtain sports performance.

In handball, the organization of the selection process aims to cover as many candidates as possible and tries to effectively use selection methods by involving the most appropriate diagnostic methods. However, for the selection to be carried out correctly and to lead to sports performance, some well-established rules must be strictly observed, some of which are listed below [23]:

- Establishing the optimal age of selection, the age at which the skills and motor qualities of the individual are obvious and have maximum efficiency in guaranteeing the achievement of performances in the chosen sport area.
- the selection criteria established to be adequate to the particularities of the sport area.
- The rejection system is recommended in the first stage of selection and training, although sometimes individuals who can achieve superior results in the future activity are rejected.
- The specific selection tests within the sport area will be applied in ascending order, according to the individual’s age, gender, development level, and training particularities.
- The selected individuals must conform well to the regional sport particularities, the sporting tradition, the training conditions, and sports facility, and with the social, economic, and professional environment conditions, which may lead to improvement of motor skills and abilities.

The enhancement of the skills and qualities of future athletes can be achieved through an extensive instructive educational process called sports training [24]. The success of this extensive training process is determined by goal establishment and choosing the right content, methods, and techniques of learning and assessment, which leads to capitalizing on the physical, mental, and biological potential of the individual. Some researchers define and describe sports training in terms of its general components, such as physical, technical–tactical, intellectual, mental, and moral training, which is a complex and planned process. From a biomedical point of view, this process aims to evolve the individual’s skills according to the objective of morphological and functional adaptation. However, physical training is the most important of all the training components. This implies the increase in the physiological potential of the individual as well as the development of the basic motor qualities: speed, skill, strength, endurance, mobility, and suppleness, and more specific ones.

At the same time, modern sports training is oriented towards fulfilling a set of clear training objectives:

- Increase in motor capacity.
- Harmonious physical development specific to the chosen sport area.
- Development of basic motor skills and formation of the foundation for the development of combined or specific motor skills.
- Learning the correct basic technical and tactical procedures of the practiced sport.
- Brief introduction of specialization and game tasks.

Participation in competitions with an optimal level of training.

- In preparing 10–12 year-old children for handball, many researchers believe that modern sports require, as the main components, physical, technical, tactical, theoretical, and psychological training [25,26,27,28]. Thus, some of the most important sports training objectives are listed below.
- Developing the motor capacity that can be achieved through borrowed means from other sports (athletics, gymnastics, adapted sports games, and dynamic games); these are the basis for the technical–tactical skills specific to handball development.
- Harmonious physical development, which is an objective achievable through posture and breathing exercises as well as through bilateral analytical work and execution of specific exercises with the clumsy body part.
- Development of motor qualities specific to the stage of physical development, speed, and skill; of course, the development of motor qualities, strength, and endurance is also attempted, but it is approached on a secondary level.
- Learning specific handball game technique, where the correct skills and specific technical procedures are imposed.
- Having an appropriate behavior in the training or competition activity (fair play, perseverance, punctuality, discipline, respect, collaboration), as well as outside the sports sphere, and respecting the basic principles of a balanced life (rest, nutrition, hygiene, etc.).

The optimization of the training process involves material, organizational, and medical factors, referring to the increase in the registered performances, as well as to the complexity of the training of the performance athletes.

The issue of optimizing the selection system in sports, especially in handball, is particularly important. In the reviewed literature, the authors consider the selection process to determine approximately 70% to 75% of destined-to-be-great athletes [29,30,31]. Talent must be found in a timely manner and included in a training system conducted in full harmony with the educational process.

By analyzing the ways of accomplishing the process of selection and sports training in Romania, as well as in other countries, such as France, Poland, or Spain, it was possible to draw some similarities and differences. Among the similarities were the concept of the game, the basis of selection, and the basic material conditions, as well as the age of selection for initiation in handball. At the same time, the training period from the initiation in handball to the achievement of great performances was comparable. For example, in 2019, there were approximately 55,000 professional athletes in France, compared to only 6000 professional athletes in Romania. Additionally, the financial support of the other countries was clearly superior compared to that of Romania, as well as the professional level of handball coaches that were included, who led an extremely efficient system of continuous training [32,33,34].

Nowadays, there is a decrease in the sports selection age compared to the existing model for children. This trend has developed from the need to quickly obtain sports performances and the continuous increase in the technical and tactical mastery levels, facts that determine the intensification of all aspects of training young athletes. The training of future athletes creates important problems due to the reduction in the performance period in which athletes are expected to achieve “great performance”, which requires the re-recruitment and completion of groups of athletes participating in major competitions [35].

According to Ghervan [35], the average age of recruitment to handball for men is 11.6 years old, and the average age at which they completed their first performance is 22.6 years old. On the other hand, at the 2012 London and the 2016 Rio Olympic games, the average age of the handball players (males and females) was 28 and 28.4 years old, respectively [36].

A study performed by Leuciuc [37] highlights the age at which handball players reach their maximum performance level. The study targeted the players involved in the final tournament of the Men’s EHF Champions League Final Four 2012. The conclusions were surprising: the period of maximum performance recorded for professional handball players was around 25 to 35 years old. To confirm the data from the study, the athletes were monitored at all the major handball competitions between 2003 and 2012.

The essence of the selection process is to establish a diagnosis and a prognosis regarding the future evolution of sports performance. In this process, the basic tool is represented by the test’s procedures. Numerous issues related to this approach were discussed at meetings of the European Federation for Sport and Body Activities, the main purpose of which was to standardize tests in the sports field. It was concluded that age-appropriate psychomotor tests should be used in the selection process, and it was proposed that test packages specific to different sport areas should be established [38,39].

A modern trend that allows the selection system’s optimization is that of the introduction of tests that assess coordinative abilities’ development level. Handball is a game mainly based on coordination, but in the selection tests for children in Romania, coordinative abilities are only tested through technical tests. The ability to coordinate is determined by a control process and movement regulation. They allow the control of predictable and unpredictable actions. Here, we can distinguish two categories. The first category includes the ability of general coordination because of multipurpose motor acquisition that allows the performance of motor tasks in an inventive, personal manner. The second category includes specific coordinative abilities, developed within a sports discipline, that allow the choice of tactical combinations specific to the respective sport. Generalized training of coordinative abilities is complex and requires the identification and prioritization of each component to be developed (combining ability, analytical ability, balance, orientation, rhythm, or reaction) [40,41].

Testing in the selection process is a major importance factor. Various scientific studies attest to this. In countries with a history of handball performance, various new selection strategies and various tests to facilitate the recruitment of young talents for handball have been adopted [42,43,44,45]. These studies propose some test packages that evaluate general physical development, coordinative capacity, and motor and psychic development, as well as some social aspects. 

In a study conducted in Poland, researchers proposed a test package for the selection of children for handball, which includes somatic measurements, motor tests, and tests that measure the level of coordinative abilities’ development. The latter is evaluated by tests where simple reaction time to visual stimuli, complex reaction time to visual stimuli, visual–motor coordination (a modified test involving equipment; Piórkowski), spatial orientation, ambidexterity, and perception and sense of direction are measured. The study’s conclusions show that assessing the level of development of coordinative abilities is extremely important for the selection of children for handball [42].

Numerous studies conducted in France and Canada also propose the inclusion of coordination tests in the process of selecting children for handball, in addition to those that assess general motor skills and physical development. The authors propose tests such as: 9-3-6-3-9, the peripheral vision test, reaction speed, throwing the ball to the target, zigzag running, and moving in a triangle. The conclusions of these studies also emphasize the importance of including tests for assessing coordinative abilities in test packages for the selection process of children for handball [46,47].

## 3. Materials and Methods

To achieve this important goal, a 10-question questionnaire was created and distributed individually (via email) to Romanian handball coaches using Google Forms (https://forms.gle/7atx7Goebz9o7tU67, accessed on 28 December 2021). The questions were asked and arranged so that the handball coaches’ opinions would be as useful as possible to highlight the benefits of using coordinative abilities in the selection and training process for children in handball. The methodology for the questionnaire application was strictly followed in order to not influence the obtained results. Firstly, we conducted a pilot questionnaire survey with 13 questions asked to 35 handball coaches to determine the questions’ validity and their comprehension degree; then, we elaborated a final questionnaire survey with 10 refined questions asked to 109 Romanian handball coaches. The questionnaire format was a three-to-five-point Likert scale. 

The survey questions were carefully selected and validated through a group of sport researchers before launching the form. The data protection guidelines, the legal General Data Protection Regulation (GDPR), and anonymization policies were first agreed upon by participants before starting the questionnaire. It is important to mention that the authors of this paper did not use the private information or the identity of questioned persons for any other purpose than the scientific one. The respondents were informed about the purpose of the questionnaire and about its scientific use. Finally, users were informed that their participation was voluntary, and some reminders were sent during the survey period to try to ensure an adequate participation rate.

The final 10-question questionnaire listed in Table 1 was filled by 109 handball coaches, 34 females and 75 males. The mean age was 40.71 years (SD = ±5.32) with a range of 31–51 years for female coaches and 40.3 years (SD = ±7.53) with a range of 30–62 years for male coaches. Their experience in handball coaching varied from 10 to 30 years, with 19.88 (SD = ±5.05) years of experience on average for female coaches and 19.09 (SD = ±5.26) years of experience on average for male coaches, as can be seen in Table 2. Many of them had obtained good results in national handball competitions (being coaches of national junior handball teams). They answered the questionnaire questions based on their experience, the knowledge they possessed, and their individual training lesson approach.

## 4. Results and Discussion

In this section, we depict handball coaches’ questionnaire answers, with the corresponding comments. By analyzing the answers from the pilot questionnaire, we established the necessity of the proposed approach, the importance of the topic in handball coaches’ activity, and also the importance of coordinative abilities’ development for handball.

Regarding question no. 1, “How do you appreciate the quality of Romanian selection in sports games?”, most handball coaches considered the quality of the selection process in our country as weak or very weak. Comparing the first three answer options (very good, good, and satisfactory) with the last two options (weak and very weak), less than 50% (41.17% female and 48% male coaches) were satisfied with the quality of the Romanian selection process in sports compared to 58.83% female and 52% male coaches who were not satisfied with it, resulting in F(1, 10) = 4.96, *p* < 0.03 for female coaches and F(1, 32) = 4.15, *p* < 0.0004 for male coaches in a statistical data analysis. The percentage difference between the answers of the handball coaches who checked the “good” and “satisfactory” options was relatively small compared to those who answered “weak” and “very weak”. None of the handball coaches answered that the quality of the Romanian selection process in sports was “very good”, meaning that there is a necessity to make improvements in this direction.

Most handball coaches, 53.21%, chose for the second question “How do you appreciate the quality of the Romanian handball game selection process?” the options “weak” or “very weak”. The rest of the handball coaches, 46.79%, were satisfied with the quality of the Romanian handball selection process. Comparing data from the first three answer options (very good, good, and satisfactory) with the last two options (weak and very weak), we can conclude that the analyzed data are statistically significant, resulting in F(1, 8) = 5.32, *p* < 0.05 for female coaches and F(2, 32) = 4.15, *p* < 0.0004 for male coaches. The fact that most of the handball coaches chose the “weak” and “very weak” options denotes the possibility of making improvements to the selection process in handball, and the research topic chosen by us is very important and up to date.

For the third question, “What do you consider to be the most effective selection criteria?”, most of the answers (35.29% female and 49.33% male) focused on the “motor” criterion as most important, while 32.35% of female and 37.33% of male coaches answered with the “somatic-and-functional” criterion, 14.7% of female and 10.66% of male coaches chose the “medical” criterion, 11.76% of female and 1.33% of male coaches chose the “psychological” criterion, and 5.88% of female and 1.33% of male coaches chose the “biochemical” criteria. The statistical significance obtained was F(1, 19) = 4.38, *p* < 0.01 for female coaches and F(1, 61) = 3.99, *p* < 0.003 for male coaches after comparing the first two answer options (motor and somatic and functional criteria) with the last three options (medical, psychological, and biochemical criteria). Most handball coaches considered the “motor” and “somatic-and-functional” criteria to be very important, but it is necessary to appreciate the interdependence and the influence of all selection criteria in obtaining sports performance. Handball performance is conditioned by a high level of motor quality development with a high genetic susceptibility, such as coordinative abilities and speed [48].

“Physical training” was the most common option for the handball coaches who answered the question “In your opinion, which of the sports training components occupies the most important place in sports training?”, totaling 55.82% for female and 53.33% for male coaches. A total of 23.53% of female and 30.66% of male coaches answered with the “technical training” option, and 14.7% of female and 10.67% of male coaches chose “tactical training”. A total of 2.94% of female and 2.66% of male coaches considered psychological and theoretical training to also be important. Statistical analysis data for female F(1, 23) = 4.28 and F(1, 59) = 4.00 for male coaches were obtained after comparing the first two answer options (physical and technical training) with the last three options (tactical, psychological, and theoretical training). At the age of 10–12-years old, motor skills are characterized by the prime learning years and they are at their greatest potential. Training involves basic principles that help to create a future handball player. The correct introduction and teaching of movement and basic technical skills is the main exercise. The aim at this stage is to continuously build up technical elements of handball [49]. So, even if physical training ensures the development of motor qualities such as coordination, speed, strength, and endurance, researchers report that technical training should occupy a major role in children’s training [50].

For question no. 5, “Which of the listed motor qualities play an important role in the handball athletes selection process?”, 32.35% of female and 28% of male coaches believed that “coordinative abilities” are the most important motor quality that 10–12-year-old children possess and represent an important role in the handball selection process. A total of 26.47% of female and 24% of male coaches answered “speed”, 23.53% of female and 24% of male coaches answered “strength”, 8.82% of female and 13.33% of male coaches answered “endurance”, and 8.82% of female and 10.66% of the male coaches chose “mobility” as playing an important role in handball selection. Statistical analysis data for female F(2, 22) = 3.44, *p* < 0.006 and F(1, 35) = 4.12, *p* < 0.003 for male coaches reveal that the data are statistically significant when comparing the first three options (coordinative abilities, speed, and strength) with the last two options (endurance and mobility). We could observe that most of the handball coaches, 54.13%, chose coordinative abilities and speed as skills they believed to play an important role in the selection of children for handball as those two are mainly developed during 10–12 years of age. Strength and endurance are motor qualities that will be developed in the later stages of junior training (after 14 years of age), due to hormonal and neuromuscular factors that converge, assuring much more advantageous adaptation [51].

For question no. 6, “Which of the components of coordinative abilities occupy an important role in handball players training process?”, the majority of surveyed handball coaches (79.41% of female and 62.67% of male coaches) chose “the orientation, differentiation and reaction” ability as being very important in children’s handball training process. The rest of the 20.59% female and 37.33% male coaches stated that balance and rhythm ability are important in children’s handball training. Comparing female coaches’ answers F(2, 21) = 3.47, *p* < 0.024 to F(2, 52) = 3.17, *p* < 0.0004 male answers, we can observed that the provided data have statistical relevance, taking into account the first three answer options (orientation, differentiation, and reaction ability) and the last two options (balance and rhythm ability). Orientation ability in handball is very important due to the athlete’s movement dynamic while playing. Finding the right space on posts, knowing the correct angle at which to throw the ball at the goal or the optimal moment to intercept are dependents on the orientation ability development level. Differentiation ability provides intel when interacting with adversaries or engaging in defense actions, highlighting the accuracy of the technical procedures’ execution, coordinating the segments in various situations. In defense, this ability allows the player to properly react to the adversary player’s action. Reaction ability implies an adequate response to an external stimulus, such as losing or taking the ball into possession, triggering a counterattack or when players have to make decisions and act with limited time and space.

For the question “How much importance is given to 10–12 years children coordinative abilities development in handball training lessons?”, most handball coaches (58.82% of female and 50.66% of male coaches) chose the “low” and “very low” options. The rest of the 41.18% female and 49.34% of male coaches chose the “average” and “high” options, respectively. Comparing female coaches’ data F(1, 10) = 4.96, *p* < 0.035 to F(2.52) = 4.16, *p* < 0.003 male coaches’ data, we can observe a statistical significance between the first three answer options (very high, high, and average) and the last two options (low and very low). The answers of the handball coaches allow us to extract the idea that children’s coordinative abilities’ development needs more attention from coaches during training lessons. Developing children’s coordinative abilities in handball requires varied training programs. Specific training for coordinative abilities is characterized by variation in execution methods, variation in stimuli, and variation in execution circumstances while performing as quickly and accurately as possible [52].

For question no. 8, “In which part of the handball training lesson do you approach the development of coordinative abilities?”, handball coaches mostly answered (44.11% of female and 44% of male coaches) the “preparatory part”, very closely followed by the second option, the “main part”, with 35.29% of female and 42.66% of male coaches answering, and 20.59% of female and 13.33% of male coaches answered the “final part”. Comparing F(1, 22) = 4.30, *p* < 0.04 for female coaches and F(1, 61) = 3.99, *p* < 0.0002 for male coaches, the data reveal statistical significance in the first two answer options (preparatory and main lesson part) from the last one, the ending part. The fact that many coaches chose to develop this quality in the preparatory and main part of the training lesson highlights, again, the importance given to this motor quality.

For the second to last question of the proposed questionnaire, “What percent of the handball training lesson you spent teaching coordinative abilities?”, 61.76% of female and 65.33% of male coaches assigned up to 20% of the handball training lesson to developing the coordinative abilities. A total of 38.24% female and 34.67% of male coaches went up to 40% of the handball training lesson dedicated to developing coordinative abilities, which allows them to use more methods for developing this motor quality. Statistical significance was found with F(1, 17) = 4.45, *p* < 0.003 for female and F(1, 45) = 4.06, *p* < 0.0001 for male coaches comparing the first two answer options (less than 10% and 11–20% of lesson time, respectively) with the last three options (21–30%, 31–40%, and more than 40%). For children the age of 10–12 years old, handball coaches should focus mainly on the motor qualities’ development and teaching technical handball procedures. To achieve this, they must spend enough time on handball training lessons.

The last question of the questionnaire concerned the importance of coordinative abilities’ development in the selection of children for handball. The answers to this question focused on the “very important” and “important” variants, the percentages being very close to 61.76% for female and 58.66% for male coaches, respectively, and 38.24% for female and 41.34% for male coaches for the “average” to “very low” options. Comparing data from female coaches F(1, 17) = 4.45, *p* < 0.003 and male coaches F(1, 40) = 4.08, *p* < 0.0003, we can conclude that the analyzed data are statistically significant, taking into account the first two answer options (very high and high) and the last three options (average, low, and very low). From the collected data, we can observe the importance we must give to developing these motor qualities, as well as the influence they have in the Romanian handball selection process.

## 5. Conclusions

In this paper, we analyzed the necessity of using the coordinative abilities in the handball selection process of children aged 10–12 years old using a questionnaire survey. In an attempt to summarize, in a few sentences, the answers to the questions of the questionnaire proposed in this paper, we can say that the Romanian selection process in sport games in general, and in handball in particular, should be raise their expectations level. At the same time, they considered that the development level of the coordinative abilities of 10–12 year-old children is medium to weak, and the actual workplan in the training process for the development of this quality is quite small. Most coaches do this in the preparatory or the main part of training lessons, using mostly 20% of training lesson time for developing coordinative abilities. The respondents also stressed the importance of developing coordinative abilities in the handball selection process for children.

The answers of handball coaches confirmed the fact that the (theoretical) in-depth study of the sports selection process in Romania is required, bringing practical solutions and contributions in this regard and giving the necessary importance to the development of coordinative abilities, which can contribute to the selection process.

Developing coordinative abilities involves components such as coordination, agility, speed, and accuracy of motor performance. Handball performance, by nature, requires a great deal of coordination.

## Figures and Tables

**Table 1:** Questions from the questionnaire proposed to evaluate the importance of coordinative abilities in the selection process for children in handball.

No.	Question
**1**	How would you rate the quality of Romanian selection in sports?
**2**	How would you rate the quality of the Romanian handball selection process?
**3**	What do you consider to be the most important selection criteria?
**4**	In your opinion, which of the sports training components occupies the most important place in sports training?
**5**	Which of the listed motor qualities play an important role in the selection process for handball athletes?
**6**	Which of the components of coordinative abilities occupy an important role in the training process for handball players?
**7**	How much importance is given to 10–12 year-old children’s coordinative abilities’ development in handball training lessons?
**8**	Which part of handball training lessons do you consider to be most important to the development of coordinative abilities?
**9**	What percent of handball training lessons do you spend teaching coordinative abilities?
**10**	How important is the level of coordinative ability development in the handball selection process?

**Table 2:** Summary of respondents (*n* = 109).

Gender	Years of Experience	No. of Subjects	Proportion
**Female**	10–16	8	23.53%
	17–23	19	55.88%
	24–30	7	20.59%
**Male**	10–16	22	29.33%
	17–23	34	45.34%
	24–30	19	25.33%

Data are presented using a variety of appropriate descriptive statistics, including frequencies, percentages, and mean and standard variation (SD). Obtained data were modeled using a one-way analysis of variation (ANOVA).

## Data Availability

Not applicable.

## References

[1] Sands W.A., Kavanaugh A.A., Murray S.R., McNeal J.R., Jemni M. (2017). Modern Techniques and Technologies Applied to Training and Performance Monitoring. Int. J. Sports Physiol. Perform..

[2] Rajšp A., Fister I. (2020). A Systematic Literature Review of Intelligent Data Analysis Methods for Smart Sport Training. Appl. Sci..

[3] Guerriero A., Varalda C., Piacentini M.F. (2018). The Role of Velocity Based Training in the Strength Periodization for Modern Athletes. J. Funct. Morphol. Kinesiol..

[4] Moisescu P. (2011). Developing Coordinative Skills and Their Influence on the Motive Performance of Pupils in Primary Education. Ph.D. Thesis.

[5] Russell J.J., Martindale D.C., Wang C.K., McNeill M., Lee K.S., Sproule J., Westbury T. (2010). Development of the Talent Development Environment Questionnaire for Sport. J. Sports Sci..

[6] Macnamara A., Collins D. (2011). Development and initial validation of the Psychological Characteristics of Developing Excellence Questionnaire. J. Sports Sci..

[7] Tam R., Beck K.L., Gifford J.A., Flood V.M., O’Connor H.T. (2020). Development of an Electronic Questionnaire to Assess Sports Nutrition Knowledge in Athletes. J. Am. Coll. Nutr..

[8] Calella P., Iacullo V.M., Valerio G. (2017). Validation of a General and Sport Nutrition Knowledge Questionnaire in Adolescents and Young Adults: GeSNK. Nutrients.

[9] Halson S.L., Appaneal R.N., Welvaert M., Maniar N., Drew M.K. (2022). Stressed and Not Sleeping: Poor Sleep and Psychological Stress in Elite Athletes Prior to the Rio 2016 Olympic Games. Int. J. Sports Physiol. Perform..

[10] Mendo A.H., Pollan R.R. (1996). Introducción a la Informática Aplicada a la Psicología del Deporte.

[11] Hughes M., Bartlett R. (2002). The use of performance indicators in performance analysis. J. Sports Sci..

[12] Ferrari W. (2018). Comparative Analysis of the Performance of the Winning Teams of the Handball World Championship: Senior and Junior Levels. Int. J. Sports Sci..

[13] Myer G.D., Lloyd R.S., Brent J.L., Faigenbaum A.D. (2013). How young is “too young” to start training?. ACSM’s Health Fit. J..

[14] Carolyn A.E., Pasanen K. (2019). Current trends in sport injury prevention. Best Pract. Res. Clin. Rheumatol..

[15] Nabatnikova M.I. (1982). On the content of training young athletes. Sci. Sports Bull..

[16] Manno R. (1992). Les Bases de L‘Entraínement Sportif.

[17] Baştiurea E. (2007). Handbal: Concepte, Principii şi căi de Perfecţionare ale Antrenamentului.

[18] Shuba L., Chukhlantseva N., Shuba V. (2018). Complex development of physical characteristics of 11–12-year-old boys using basketball elements for physical education. J. Phys. Educ. Sport.

[19] The XXXII FIMS World Congress of Sports Medicine. http://www.fimsroma2012.org.

[20] Juravle I. (2012). Contribution regarding handball selection using graphical analysis method. Ann. Dunarea De Jos Univ. Galati Fascicle XV.

[21] Camacho-Cardenosa A., Camacho-Cardenosa M., González-Custodio A., Martínez-Guardado I., Timón R., Olcina G., Brazo-Sayavera J. (2018). Anthropometric and Physical Performance of Youth Handball Players: The Role of the Relative Age. Sports.

[22] Tróznai Z., Utczás K., Pápai J., Négele Z., Juhász I., Szabó T., Petridis L. (2021). Talent Selection Based on Sport-Specific Tasks Is Affected by the Relative Age Effects among Adolescent Handball Players. Int. J. Environ. Res. Public Health.

[23] Simion G., Stănculescu G., Mihăilă I. (2011). Antrenament Sportiv, Concept Systemic.

[24] Till K., Baker J. (2020). Challenges and [possible] solutions to optimizing talent identification and development in sport. Front. Psychol..

[25] Altavilla G. (2019). Monitoring training to adequate the teaching method in training: An interpretative concepts. J. Phys. Educ. Sport.

[26] Nuriddin N. (2021). The main concepts and current problems of the preparing system of runners in modern sport theory. Cent. Asian J. Theor. Appl. Sci..

[27] Kaufman K.A., Glass C.R., Pineau T.R. (2018). Mindful Sport Performance Enhancement: Mental Training for Athletes and Coaches.

[28] Maksymchuk B., Matviichuk T., Surovov O., Stepanchenko N., Opushko N., Sitovskyi A., Kosynskyi E., Bogdanyuk A., Vakoliuk A., Solovyov V. (2020). Training Future Teachers to Organize School Sport. Rev. Rom. Pentru Educ. Multidimens..

[29] Marczinka Z. (2011). What’s the difference? Coaching female and male handball players. EHF Sci. Conf..

[30] Moisescu P. (2010). Determining the optimal period in developing the coordinative capacities in male elementary school pupils. Ann. Univ. Dunărea De Jos Galați Fascicle XV Phys. Educ. Sport Manag..

[31] Increasing the Efficiency of Training, Communications, Technical-Methodical Commission. https://frh.ro.

[32] Leuciuc F. (2018). Longitudinal study on the effectiveness of the game actions in men’s handball top competitions (1998–2016). Kinesiol. Slov..

[33] Massuça L.M. (2011). Expertise evaluation of technical and tactical proficiency in handball: Differences between playing status. EHF Sci. Conf..

[34] Prisăcaru I.R., Marinescu G. (2010). Analysis of tactics and offensive playing systems in elite European teams, in vue of optimizing attack for Romanian top handball male teams. Discobolul—Rev. UNEFS De Cult. Educ. Sport Şi Kinetoterapie.

[35] Ghervan P. (2014). Selecția și Primii Pași în Handbal.

[36] Wood R. (2015). Anthropometry for Handball. https://www.topendsports.com/sport/handball/anthropometry.htm.

[37] Leuciuc F.V. (2012). Somatic Model and Performance Age in Elite Handball (Study Case: Male Champions League Final Four 2012). Gymn. Sci. J. Educ. Sports Health.

[38] Monea D., Ormenisan S., Grosu E., Apostu P., Nica C., Badea D. (2015). Optimizarea procesului de selecție în jocul de fotbal la copii în vârstă de 10-12 ani. Rev. Studia Univ. Babeș-Bolyai.

[39] Karabatic L. L’entretien du Lundi, je Suis un Hansballeur. www.ff-handball.org.

[40] Swiss Olympic (2015). Manuel de Diagnostic de Performance. https://www.swissolympic.ch.

[41] Lidor R., Falk B., Arnon M., Cohen Y., Segal G., Lander Y. (2005). Measurement of talent in team handball: The questionable use of motor and physical tests. J. Strength Cond. Res..

[42] Zubik M., Spieszny M., Sumara M. (2013). Identifying talented handball players—The possibilities of examining the players by means of speed-force and coordination tests. Cent. Eur. J. Sport Sci. Med..

[43] Machnacz W., Dudkowski A., Rokita A. (2013). Dependecies between the methods used in identifying player talent in the game of handball. Univ. Sch. Phys. Educ. Wrocław.

[44] Moss S.L., McWhannell N., Michalsik L.B., Twist C. (2015). Anthropometric and physical performance characteristics of top-elite, elite and non-elite youth female team handball players. J. Sports Sci..

[45] Wrang C.M., Rossing N.N., Diernæs R.M., Hansen C.G., Dalgaard-Hansen C., Karbing D.S. (2018). Relative age effect and the re-selection of Danish male handball players for national teams. J. Hum. Kinet..

[46] Marsh J.L. (1972). A Study of Predictability of Ability in Handbal. Master’s Physical Education Thesis.

[47] Leveque M. (2005). Psychologie du Metier D’entraineur.

[48] Galaftion C. (2004). Considerente privind importanta selectiei in obtinerea performantelor sportive. Anu. Acad. Terest..

[49] European Handball Federation (1997). The Steps to Handball. http://activities.eurohandball.com/hb4all/content/2BasicHB/TheStepstoHandball.pdf.

[50] Kunst-Ghermănescu I., Gogaltan V., Jianu E., Negulescu I. (1983). Teoria și Metodica Handbalului.

[51] Schultz R. How Young Is Too Young to Start Lifting Weights?. https://www.mensjournal.com/health-fitness/how-young-too-young-start-lifting-weights.

[52] Hasballa A.M.Z. (2010). Variance as base for training coordinative abilities and its effect on developing some defensive moves for handball beginners. World J. Sports Sci..

